# Effects of different cytokines on immune responses of rainbow trout in a virus DNA vaccination model

**DOI:** 10.18632/oncotarget.23095

**Published:** 2017-12-11

**Authors:** Yongsheng Cao, Qiya Zhang, Liming Xu, Shaowu Li, Di Wang, Jingzhuang Zhao, Hongbai Liu, Jian Feng, Tongyan Lu

**Affiliations:** ^1^ Heilongjiang River Fishery Research Institute of Chinese Academy of Fishery Sciences, Harbin, China; ^2^ State Key Laboratory of Freshwater Ecology and Biotechnology, Institute of Hydrobiology, Chinese Academy of Sciences, Wuhan, China; ^3^ Benxi Agrimarine Company Limited, Benxi, China

**Keywords:** rainbow trout, cytokines, adjuvant, DNA vaccine, protection, Immune response, Immunology

## Abstract

Seven rainbow trout cytokine genes (interleukin (IL)-2, IL-8, IL-15, IL-17, IL-1β, intracellular interferon (iIFN) 1a, and IFN-γ2) were evaluated for their adjuvant effects on a DNA vaccine, called pG, containing the glycoprotein gene of infectious hematopoietic necrosis virus (IHNV). Distinct DNA constructs in expression plasmid pcDNA3.1 encoding a cytokine gene were generated. Immunofluorescence assays in rainbow trout gonadal cells demonstrated successful protein expression from all these constructs. Subsequently, fish were immunized with pG alone or together with a cytokine expression plasmid. Results showed that each cytokine plasmids at an appropriate dose showed notable effects on immune gene expression. IL-17 and IFN-γ2 can enhance early specific IgM response. All cytokines, except IL-8, can benefit initial neutralizing antibody (NAb) titers. At 35 days post immunization (dpi), NAb titers of fish immunized with pG and IL-2, iIFN1a, or IFN-γ2 plasmids remained at high levels (1:160). NAb titers of fish immunized with pG alone decreased to 1:40. IL-8 or IL-1β can enhance antigen-specific proliferative T-cell responses at 14 dpi. At 28 dpi, coinjection of pG with IL-2, IL-8, IL-15, or IL-17 plasmids induced considerably stronger lymphocyte proliferation than that with injection of pG alone. All cytokine plasmids delivered with pG plasmid enhanced protection of trout against IHNV-mediated mortality. These results indicate that the type and dose of trout cytokine genes injected into fish affect quality of immune response to DNA vaccination.

## INTRODUCTION

Fish and aquaculture remain important sources of food, nutrition, income, and livelihood for many people worldwide. Rainbow trout (*Oncorhynchus mykiss*) is an important component of commercially valuable cold-water fish species in many countries [1]. However, rainbow trout production may suffer from severe losses due to infectious disease outbreaks [2, 3]. Vaccine is the most appropriate method to prevent accidental effects of pathogenic microorganisms on rainbow trout [4, 5].

Inactivated or genetic engineering fish vaccines are developed for their biosafety, but they cannot elicit desired protective immune responses in some cases [6, 7]. Injection is currently a major immune route for fish vaccination, but it is laborious and inapplicable in larval fish, which are susceptible to pathogens [8]. According to the economic view, oral vaccination is the ideal route for vaccination programs, which require one or more booster immunizations [9]. Nevertheless, oral delivery antigen alone commonly results in low efficacy and short protection period [10]. Numerous studies proved that additional adjuvants in fish vaccines can enhance potency and longevity of specific immune responses to antigens [11, 12].

Cytokines are naturally low-molecular weight and secretory proteins that are produced in response to infection, and they are closely associated with immune system regulation [13]. In fish, the major cytokine families are present and expressed at the transcript level [14]. Fish cytokines are not properly used as vaccine adjuvants compared with their counterparts that are applied for the same purpose in mammals owing to deficient immunological roles [15]. Nonetheless, increasing numbers of studies reported that these molecules participate similarly to regulation of fish immune responses compared with those of mammals [16, 17]. Type I interferon (IFN-I), interleukin (IL)-1β, or IL-8 present conserved adjuvant effects on inactivated or DNA vaccine in fish [18–20].

To date, modulated immune responses by cytokines are not considerably understood compared with those of existing cytokine genes in fish. Abilities of various adjuvants to enhance immune responses induced by vaccines should be also determined and compared in a single system; results of such comparison will benefit elimination of impulsive interferences [21]. Infectious hematopoietic necrosis virus (IHNV) is a disease agent in salmonids [22]. In our previous work, we generated a DNA vaccine, named as pG, with the glycoprotein (G) gene of a Chinese IHNV isolate Sn1203 based on the pEE12.4 vector. However, pG fails to provide desired protective effects. In the present study, seven types of rainbow trout cytokines, namely, IL-2, IL-8, IL-15, IL-17, IL-1β, intracellular IFN1a (iIFN1a), and IFN-γ2, were selected to investigate their effects on pG immune responses, including immune-related gene expression, serum-specific antibody level, neutralizing antibody (NAb) titer, lymphocyte proliferation, and protection. To our knowledge, this study is the first to collectively illustrate adjuvant effects of these cytokine genes of rainbow trout *in vivo*. This work will benefit screening of novel vaccine adjuvants for rainbow trout and provide valuable insights into trout immune responses.

## RESULTS

### Plasmid construction, protein expression, and polyclonal antibody preparation

**Figure 1:**
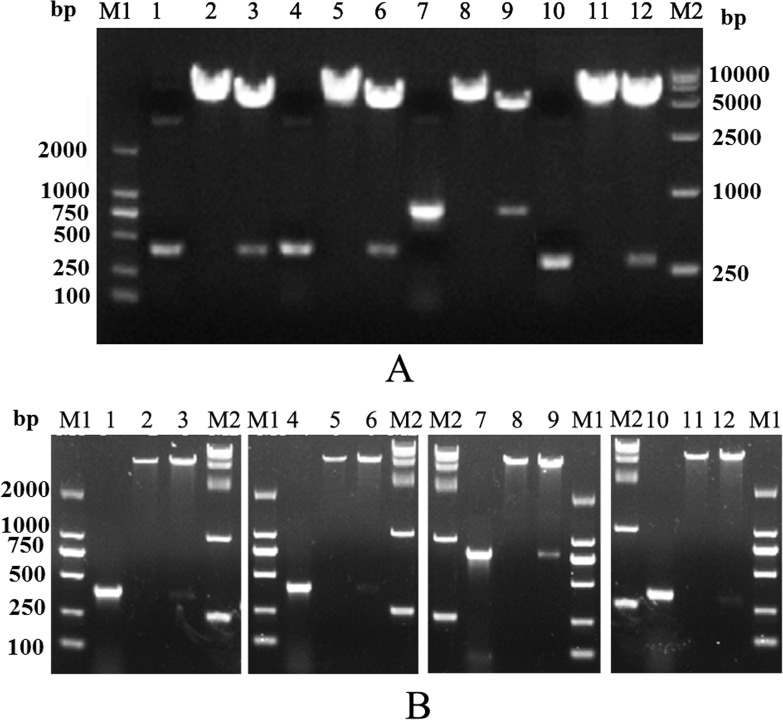
Construction of recombinant plasmids (**A**) Identification of recombinant eukaryotic plasmids. (**B**) Identification of recombinant prokaryotic plasmids. M1, Trans 2K DNA marker; M2, Trans 15K DNA marker; lanes 1–3, PCR and digestion of recombinant plasmids containing IL-2 gene; lanes 4–6, PCR and digestion of recombinant plasmids containing IL-15 gene; lanes 7–9, PCR and digestion of recombinant plasmids containing IL-1β gene; lanes 10–12, PCR and digestion of recombinant plasmids containing IL-8 gene.

Target DNA fragments were successfully amplified and cloned into pcDNA3.1 or pET32a plasmids. Polymerase chain reaction (PCR) and digestion proved that the recombinant plasmids contained cytokine DNA fragments (Figure 1A and 1B). Additionally, accuracies of fragments were confirmed by sequencing (data not shown). We named these eukaryotic expression plasmids as pIL-2, pIL-8, pIL-15, pIL-17, pIL-1β, piIFN1a, and pIFN-γ2.

**Figure 2:**
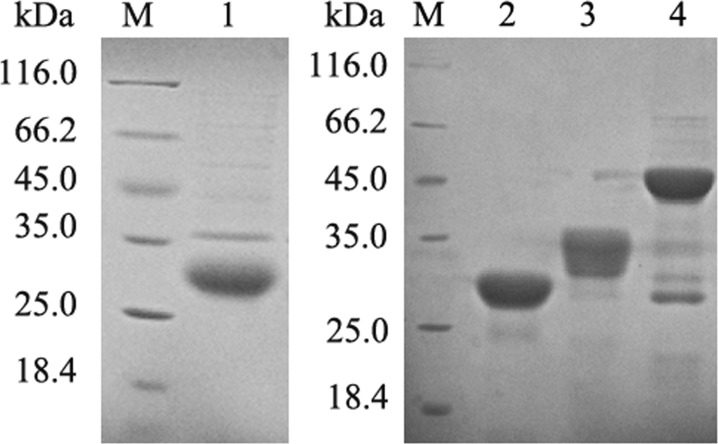
Expression and purification of recombinant proteins M, unstained protein marker; lanes 1–6, purified recombinant IL-2, IL-8, IL-15, and IL-1β proteins, respectively.

After induction with isopropyl β-D-1-thiogalactopyranoside (IPTG), *Escherichia coli* cells containing recombinant prokaryotic expression plasmids successfully expressed recombinant proteins, which were mostly located in inclusion bodies. In purification of His-tagged proteins under denaturing conditions in the Qiagen handbook, target proteins were purified at 33.7, 30.8, 34, and 46 kDa with recombinant proteins of IL-2, IL-8, IL-15, and IL-1β, respectively (Figure 2). Recombinant proteins of IL-17, iIFN1a, and IFN-γ2 were prepared and characterized in our previous work [23–25].

**Figure 3:**
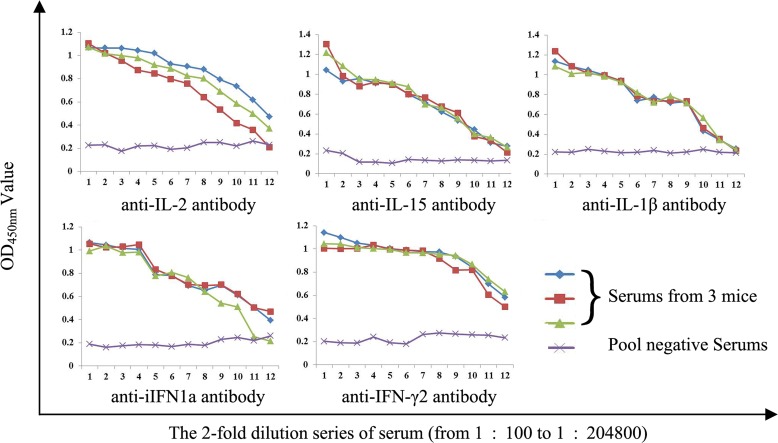
Determination of antiserums titers by indirect ELISA

After immunizing mice with purified proteins for four times, final titers of polyclonal antibodies against corresponding cytokines were determined by indirect enzyme-linked immunosorbent assay (ELISA). Results showed that titers of these polyclonal antibodies were all above 1:25600 (Figure 3). Thus, these polyclonal antibodies can be used for the following detection of expressions of cytokine genes in rainbow trout gonadal (RTG-2) cells.

### Expression of cytokine genes in RTG-2 cells

**Figure 4:**
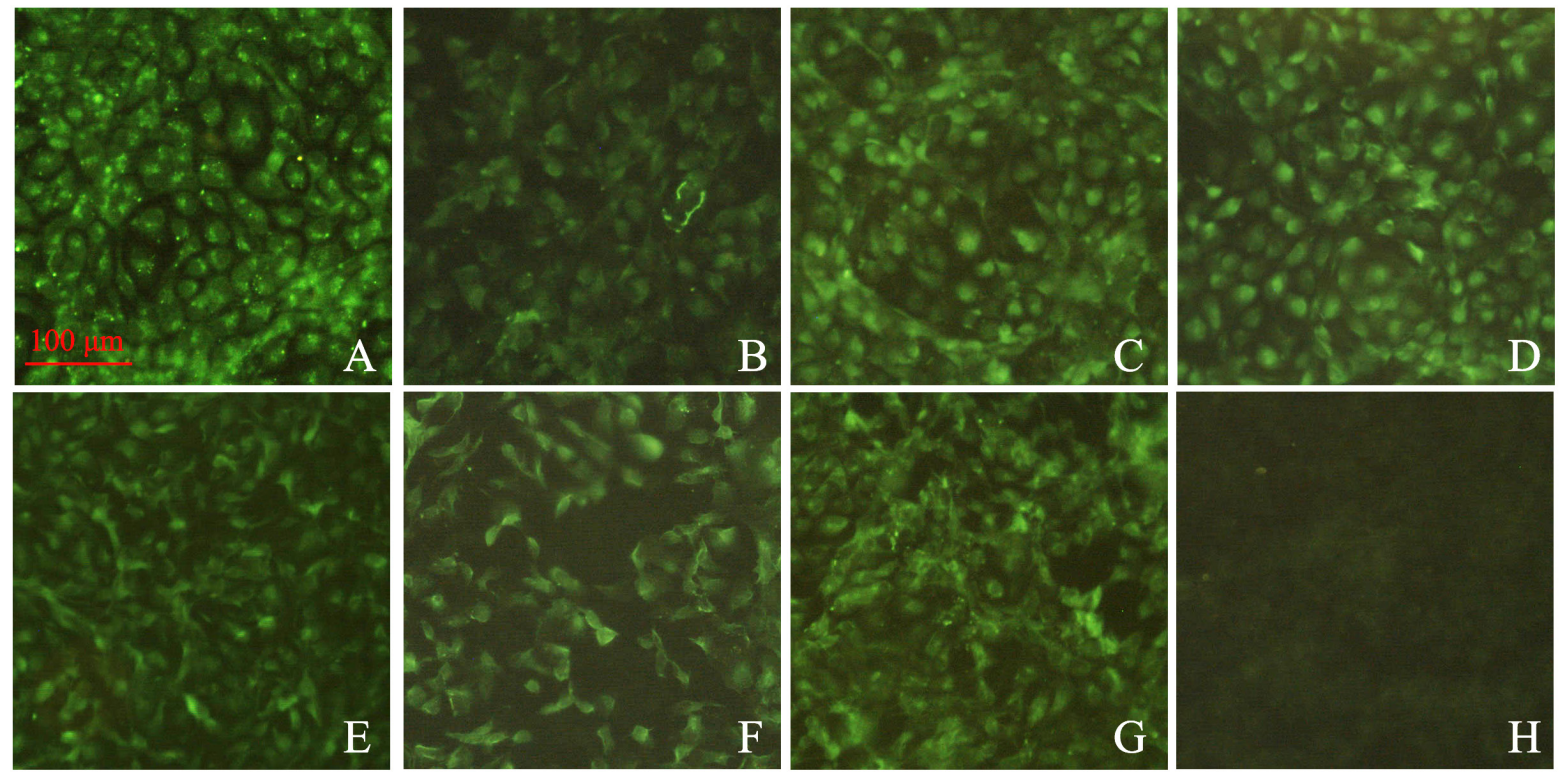
Immunofluorescence analysis of RTG-2 cells transfected with recombinant plasmids containing the cytokine gene **A**–**G**. Cells were transfected with pcDNA3.1-IL-2, IL-8, IL-15, IL-17, IL-1β, iIFN1a, and IFN-γ2, respectively. Subsequently, transient expressions of proteins within cells were detected with the corresponding polyclonal antibodies. **H**. Cells were transfected with pcDNA3.1 empty vector, which served as negative control. The green signals reflect positive expressions of proteins of interest.

RTG-2 cells were transfected with recombinant pcDNA3.1 plasmids containing cytokine genes. At 48 h post-transfection, cells were stained and incubated with the corresponding poly antiserums and fluorescein isothiocyanate (FITC)-conjugated anti-mouse IgG antibody. Specific fluorescence signal can be observed in cells transfected with pcDNA3.1-cytokines, whereas no specific fluorescence signal was observed in cells transfected with the pcDNA3.1 vector (Figure 4). Expression of pcDNA3.1-IL-17 in RTG-2 cells was confirmed in our previous work [23]. Results clearly show that the seven types of cytokine genes can be expressed from recombinant pcDNA3.1 plasmids in RTG-2 cells.

### Effect of cytokine plasmid coinjection on expressions of immune-related genes

**Figure 5:**
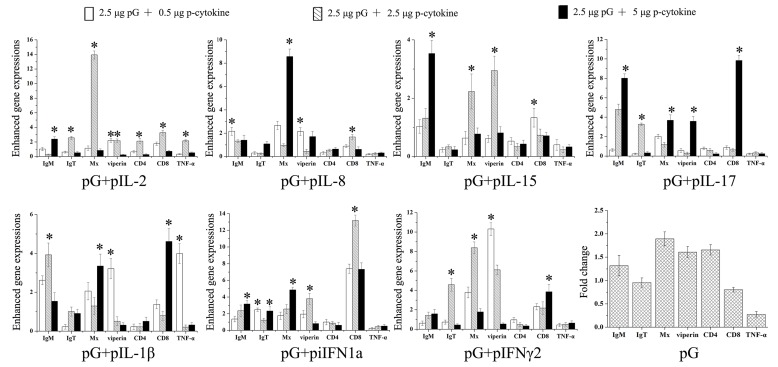
Enhanced immune-related gene expression in spleens of rainbow trout Five fish were intraperitoneally injected with pG alone or pG with cytokine adjuvants (0.5, 2.5, or 5 μg). At 3 dpi, expressions of immune-related genes in the spleen were analyzed with real-time PCR. Fold changes in immune gene expression in adjuvant groups were calculated relative to that in pG group. *, P<0.05.

Fold changes in immune-related gene expression induced by three distinct doses of cytokine plasmid were analyzed with real-time PCR and compared (Figure 5). Results showed that, especially expressions of Mx and viperin, coinjection with any type of cytokine plasmid at a proper dose can enhance the majority of immune-related gene expressions compared with the injection with pG alone. Furthermore, these cytokines exert distinct effects on immune response. For example, IL-2 showed overall enhancements on the tested genes compared with those of other cytokines. IL-17 can enhance expressions of IgM and CD8 evidently compared with those of others. Diversity effects of one cytokine depend on injection dose. Although any dose can enhance IgM expression, IL-1β can maximize this action at 2.5 μg injection. IgT expression can only be modulated by IFN-γ2 at 2.5 μg injection. To guarantee maximum and general adjuvant effects of cytokines, cytokine plasmids were injected at a dose that can enhance the most immune-related gene expression induced by pG alone. In this study, IL-2, IL-8, IL-15, IL-17, IL-1β, iIFN1a, and IFN-γ2 plasmids were injected at 2.5, 0.5, 2.5, 5.0, 0.5, 5.0, or 2.5 μg in the following immunization, respectively.

### Effect of cytokine plasmid coinjection on production of specific antibodies

**Figure 6:**
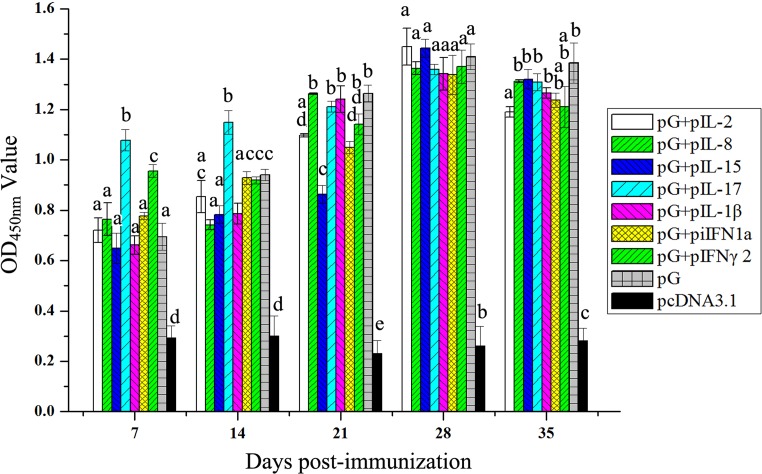
Anti-infectious IHNV-G IgM levels in fish treated with pG and cytokine DNA Fish were immunized with pG (2.5 μg) alone or pG with IL-2 plasmid (2.5 μg), IL-8 plasmid (0.5 μg), IL-15 plasmid (5 μg), IL-17 plasmid (2.5 μg), IL-1β plasmid (0.5 μg), iIFN1a plasmid (5 μg), IFN-γ2 plasmid (2.5 μg), or cDNA3.1 plasmid. Serum samples were collected each week of post immunization until 35 dpi. Anti-IHNV-G IgM antibodies were detected with indirect ELISA. Different letters indicate significant differences (P<0.05).

To evaluate the efficiency of cytokine adjuvants to enhance antibody response of rainbow trout against IHNV antigen, the fish were injected intramuscularly with pG alone or pG with cytokine plasmid or pcDNA3.1 plasmid. During each week of post immunization, sera were harvested, and specific IgM antibody was measured by ELISA using the recombinant IHNV-G protein as antigen (Figure 6). In general, levels of specific IgM in fish immunized with pG alone or together with cytokine plasmid were significantly higher than those receiving pcDNA3.1 (P<0.05). At 7 days post immunization (dpi), specific IgM levels induced by the combination of pG and IL-17 or IFN-γ2 plasmid were significantly higher than that induced by pG alone (P<0.05). Furthermore, the combination of pG and IL-17 plasmid induced significantly higher specific IgM than pG alone at 14 dpi (P<0.05). However, with increasing IgM levels, no significant differences were observed in levels of specific IgM present in the fish immunized with pG and combination of pG and IL-8, IL-17, IL-1β, or IFN-γ2 plasmid. Unexpectedly, specific IgM levels in the remaining three adjuvant groups were significantly lower than those in the pG group (P<0.05). At 28 dpi, specific IgM reached the highest level, and no significant differences were observed among pG or adjuvant groups. Afterward, specific IgM levels decreased between 28 and 35 dpi. During this period, only the specific IgM level induced by the combination of pG and IL-2 plasmid was significantly lower than that induced by pG alone (P<0.05).

### Effect of cytokine plasmid coinjection on NAb titers

Sera from three individual fish in all treated groups were collected and pooled. NAb titers were determined using plaque neutralization titer (PNT) procedure for IHNV (Table 2). At 7 dpi, only the combination of pG and IL-17, iIFN1a, or IFN-γ2 plasmid induced positive NAb titers (1:40). At 14 dpi, the fish showed positive NAb titers, except for those injected with pG alone or co-injected with pG and IL-8 or IL-1β plasmid. At the following dpi, fish in experimental groups all displayed positive NAb titers with respective dynamics. NAb titers of fish treated with the combination of pG and IL-2 or IL-15 plasmid reached the highest level at 28 dpi (1:160) and lasted until or decreased to 1:80 at 35 dpi. NAb titers of fish treated with the combination of pG and IL-8 or IL-1β plasmid also reached the highest level at 28 dpi (1:80 or 1:160) and decreased to 1:40 or 1:80 at 35 dpi. NAb titers of fish treated with the combination of pG and IL-17 plasmid reached the highest level at 21 dpi (1:160), decreased at 28 dpi, and persisted until 35 dpi (1:80). Additionally, NAb titers of fish treated with the combination of pG and iIFN1a or IFN-γ2 plasmid reached the highest level at 21 or 14 dpi and lasted until 35 dpi (1:160). NAb titers of fish treated with pG alone reached the highest level at 21 dpi (1:160) and decreased to 1:80 and 1:40 at 28 and 35 dpi, respectively. No NAbs were detected in sera from pcDNA3.1-treated fish.

**Table 1:** Primers used for cloning, expression and qRT-PCR of genes in this study

Primer name	Primer sequences (5' to 3')	Application
IL-2 *Bam*H I-F	GGATCCAACCCAATTCCCAGACTCCT	prokaryotic expression plasmid construction
IL-2 *Hind* III-R	AAGCTTTTATGAACTTAGACGCTTTGC	
IL-2 *EcoR* I-F	GAATTC**GCCACC**ATGAACCCAATTCCCAGACTCCT	eukaryotic expression plasmid construction
IL-2 *Xho* I-R	CTCGAGTCATGAACTTAGACGCTTTGC	
IL-8 *BamH* I-F	GGATCCATGAGCATCAGAATGTCAGCCAG	prokaryotic expression plasmid construction
IL-8 *Hind* III-R	AAGCTTTTATTTGTTGTTGGCCAGCATCT	
IL-8 *EcoR* I-F	GAATTC**GCCACC**ATGAGCATCAGAATGTCAGCCAG	eukaryotic expression plasmid construction
IL-8 *Xho* I-R	CTCGAGTCATTTGTTGTTGGCCAGCATCT	
IL-15 *BamH* I-F	GGATCCGCTGAAACACACGGGATGA	prokaryotic expression plasmid construction
IL-15 *Hind* III-R	AAGCTTTTAACTGACAGTTTGCCCTATTC	
IL-15 *EcoR* I-F	GAATTCGCCACCATGGCTGAAACACACGGGATGA	eukaryotic expression plasmid construction
IL-15 *Xho* I-R	CTCGAGTCAACTGACAGTTTGCCCTATTC	
IL-17 *EcoR* I-F	GAATTC**GCCACC**ATGGAGCTCAAAAGCAACGT	eukaryotic expression plasmid construction
IL-17 *Xho* I-R	CTCGAGTCAAGTAGTCCTTGCCCA	
IL-1β *EcoR* I-F	GAATTCATGGATTTTGAGTCAAACTAC	prokaryotic expression plasmid construction
IL-1β *Hind* III-R	AAGCTTTTAGTTGTGGCGCTGGATGGT	
IL-1β *EcoR* I-F	GAATTCGCCACCATGGATTTTGAGTCAAACTAC	eukaryotic expression plasmid construction
IL-1β *Xho* I-R	CTCGAGTCAGTTGTGGCGCTGGATGGT	
iIFN1a *EcoR* I-F	GAATTC**GCCACC**ATGCAGAGCGTGTGTCATTG	eukaryotic expression plasmid construction
iIFN1a *Xho* I-R	CTCGAGTCAGTACATCTGTGCCGCAA	
IFN-γ2 *EcoR* I-F	GAATTC**GCCACC**ATGGCTCAGTACACATCAATTAAC	eukaryotic expression plasmid construction
IFN-γ2 *Xho* I-R	CTCGAGTCACATGATGTGTGATTTGAG	
IgM F	CAAACCGGTGGAAGCTACAT	real time PCR
IgM R	AGACGGCTGCTGCAGATATT	
IgT F	AACATCACCTGGCACATCAA	real time PCR
IgT R	TTCAGGTTGCCCTTTGATTC	
Mx F	AGCGTCTGGCTGATCAGATT	real time PCR
Mx R	AGCTGCTCGATGTTGTCCTT	
Viperin F	GTGTTCCAGTGTCTGCTGATCGAT	real time PCR
Viperin R	TGATGCTGCTGTGCCTTTCC	
CD4 F	CATTAGCCTGGGTGGTCAAT	real time PCR
CD4 R	CCCTTTCTTTGACAGGGAGA	
CD8 F	GACTGCTGGCTGTGGCTTCC	real time PCR
CD8 R	CCCCGGAGCTGCCATTCT	
TNF-α F	TCTTACCGCTGACACAGTGC	real time PCR
TNF-α R	AGAAGCCTGGCTGTAAACGA	
β-Actin F	GCCGGCCGCGACCTCACAGACTAC	real time PCR
β-Actin R	CGGCCGTGGTGGTGAAGCTGTAAC	

Underlined nucleotides are restriction sites, bold shown as Kozak consensus sequence.

**Table 2:** The detection of NAb titers

Dpi Groups	7	14	21	28	35
pG+pIL-2	1:20	1:40	1:80	1:160	1:160
pG+pIL-8	1:20	1:20	1:40	1:80	1:40
pG+pIL-15	<1:20	1:40	1:80	1:160	1:80
pG+pIL-17	1:40	1:40	1:160	1:80	1:80
pG+pIL-1β	1:20	1:20	1:20	1:160	1:80
pG+piIFN1a	1:40	1:80	1:160	1:160	1:160
pG+pIFN-γ2	1:40	1:160	1:160	1:160	1:160
pG	1:20	1:20	1:160	1:80	1:40
pcDNA3.1 plasmid	<1:20	<1:20	<1:20	<1:20	<1:20

Fish were immunized with pG alone or together with cytokine plasmid or pcDNA3.1. Serums of three fish from each group were collected and pooled after each week of post immunization. Titers <1:20 were defined as negative.

### Effect of cytokine plasmid coinjection on lymphocyte proliferation

**Figure 7:**
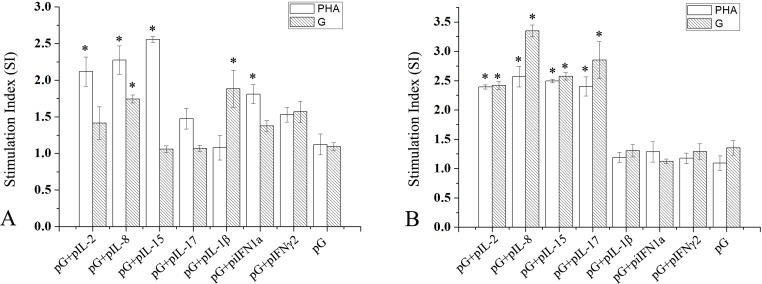
Proliferative T-cell responses in spleens of immunized fish Fish were immunized with pG alone or together with cytokine plasmid or pcDNA3.1 plasmid. At 14 and 28 dpi (**A** and **B**), leukocytes from spleens of three rainbow trout from each experimental group were isolated and cultured under PHA or recombinant G protein stimulation. Stimulation indices between each group were compared. *, P<0.05.

At 14 and 28 dpi, lymphocytes from three fish in each experiment group were cultured with phytohaemagglutinin (PHA) or recombinant IHNV-G protein. Effects of PHA and specific antigen on lymphocyte proliferation were determined (Figure 7). At 14 dpi, PHA exhibited significant stimulatory effects on cells from fish co-immunized with pG and IL-2, IL-8, IL-15, or iIFN1a plasmids compared with fish immunized with pG alone or pG and IL-17, IL-1β, or IFN-γ2 plasmid (P<0.05). Additionally, lymphocytes from fish co-injected with pG and IL-8 or IL-1β plasmid proliferated significantly under the stimulation of recombinant IHNV-G protein compared with those from fish in other groups (P<0.05). However, at 28 dpi, both PHA and recombinant IHNV-G protein showed significant stimulatory effects on cells from fish co-immunized with pG and IL-2, IL-8, IL-15, or IL-17 plasmid compared with those of fish immunized with pG alone or pG and IL-1β, iIFN1a, or IFN-γ2 plasmid (P<0.05).

### Cytokines increase protection of pG against IHNV challenge

**Figure 8:**
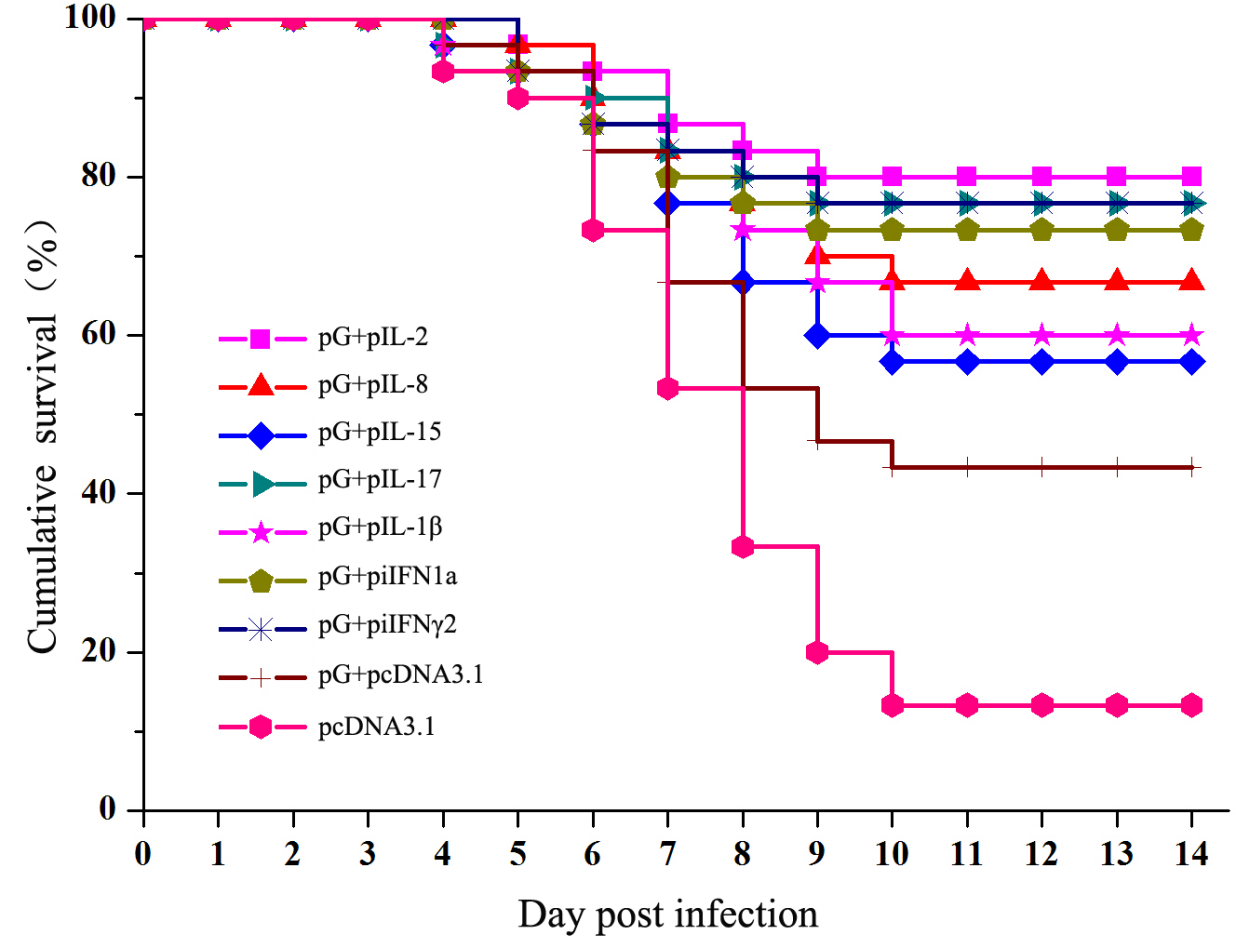
Survival plots for vaccinated trout after being challenged with IHNV Fish were injected with intramuscular injection of pG alone or the combination of pG and cytokine plasmids. At 28 dpi, fish were challenged by intraperitoneal injection with 300 pfu of IHNV.

To assess whether cytokine may increase protective effects of pG against IHNV infection, immunized fish were challenged with IHNV at 28 dpi, and accumulated fish survivals were determined. At 14 days after injection of IHNV, fish injected with pG alone showed significantly higher protection (43.3% survival) than those injected with pcDNA3.1 (13.3% survival). Coinjection with any cytokine plasmid resulted in significant protection. Among the cytokine plasmids used, IL-2, IL-17, iIFN1a, and IFN-γ2 plasmid strongly enhanced protection (80%, 76.7%, 73.3%, and 76.7% survival, respectively). Survival resulting from coinjection of pG with IL-8 and IL-1β plasmids reached 66.7% and 60%, respectively. IL-15 displayed weak adjuvant effects on protection (56.7% survival) (Figure 8).

## DISCUSSION

Among genetic engineering vaccines, DNA vaccines containing protective antigens in recombinant plasmids are relatively efficient in inducing protective immune response against fish rhabdoviruses in salmons [26]. However, DNA vaccines against infectious pancreatic necrosis virus, infectious salmon anemia virus, or lymphocystis disease virus are less effective in protection [27]. Additionally, many aspects, such as alternative routes of immunization that allow mass or fry vaccination, should still be optimized with adjuvants [10, 28]. Therefore, rational fish vaccines should be designed; these vaccines must include a combination of antigens and tailored adjuvant systems that induce effective immune responses against specific pathogens without adverse side effects [15].

Adjuvants should be used at a proper dose because low dose of some adjuvants can provide similar enhancement effects [29]; at high doses, adjuvants promote opposite, tolerogenic responses [30]. Fish cytokines are parts of the animals’ natural responses to microbial infection or vaccination [31]. Nevertheless, only few studies have given considerable attention to antigen/cytokine adjuvant ratio in fish vaccine. Immune relevant gene expression analyses are widely recognized as essential for determination of fish immune responses. Gene expression induced with antigens at different doses of seven different trout cytokine plasmids were investigated for the first time. Different doses of cytokine plasmids showed remarkable adjuvant effects on induction of immune genes. Fish vaccine preparation commonly includes construction of expression plasmid containing antigens and cytokine gene or mixture of antigens with equal volumes of cytokine [32, 33]. According to our results, IL-2, IL-15, and IFN-γ2 are suitable as adjuvants when used in this manner. Nonetheless, IL-17 and iIFN1a may function significantly at high doses. Although the knowledge on trout IL-17 remains limited, dose–response studies on human IL-17-induced IL-1β and tumor necrosis factor (TNF)-α release showed that IL-1β and TNF-α release increases with IL-17 concentration, and an effective concentration is necessary to increase their release [34]. iIFN1a is a member of trout IFN-a, and it serves as a functional iIFN that performs novel defense mechanism to combat viral infections [35]. This distinct function characteristic may have resulted in high dose need of iIFN1a to stimulate efficient immune response. IL-8 of rainbow trout and other fish can modulate early immune responses against infections [33, 36]. Our results indicate that low IL-8 concentration may provide better stimulus than at high concentrations. IL-1β also exerted significant effects on immune response at low doses, agreeing with previous results showing that the specific IgG induced with low dose of recombinant mouse IL-1β is comparable to that induced with normal dose [29].

An effective immune response to immunization should include the innate and adaptive immune subsystems [37]. Innate immunity plays important roles in the first defense and a crucial part in initiation and subsequent direction of adaptive immune responses [38]. IHNV G protein is a good inducer of IFN-I [39]. In our study, cytokine plasmids further up-regulated expressions of antiviral proteins (Mx and viperin). Both our previous study and other's work proved that antiviral abilities of trout IFNs depend on dose and kinetics of antiviral state [24, 40]. Hence, cytokine plasmids may enhance efficiency and longevity of nonspecific protection before the initial adaptive immune response takes effect. However, challenge experiments in early stages of immunization should be performed to ascertain this hypothesis.

Long-term stimulation of humoral and cell-mediated arms of the adaptive system must be provided by immunization to combat virus infection. Mammalian cytokines are immune potentiators that target the innate immune system [37]. Based on bioactivities of many trout cytokines, future studies should examine the possibility of improving adaptive immunity with various trout cytokines. To analyze the onset of humoral response, rainbow trout was immunized with combinations of pG and cytokine plasmids. Only IL-17 and IFN-γ2 can significantly enhance early specific IgM response. All these cytokine genes exerted no contribution to the peak level of specific IgM induced by pG. This phenomenon was also observed in a former study. Two immunizations of DNA vaccine in combination with any of immunomodulator expression vectors (IL-2, IL-12, IL-15, or granulocyte-macrophage colony-stimulating factor) exhibit no significant alteration on serum antibody response observed in immunized mice; however, a third immunization results in further increase in specific antibody titer in these mice [41]. Enhanced immunization with the combination of pG and cytokine plasmid may have caused this significance. Given that the NAbs are both sufficient and necessary for protection against viral infections [42], we evaluated whether cytokine plasmids cause differences in NAb production. All the tested cytokines, except IL-8 and IL-1β, can enhance early response and longevity of NAbs. Thus, effects of cytokine gene injection on humoral response induced by pG mainly relies on NAb production. Coinjection of IL-8 plasmid with DNA vaccine modulates cytokine response of rainbow trout mainly by promoting the increase in proinflammatory cytokines (IL-1β and TNF-α) [36]. Our results indicate that IL-8 was less effective in inducing NAb response, whereas IL-1β exerted no effect on early production of NAbs. Nonetheless, fish IL-8 enhances antibody response in DNA vaccination against bacterial infection [20]. Considering that limited available knowledge about adjuvant effects of fish IL-8 in virus DNA vaccination can be used for comparison, difficulty arises from determining causes resulting in less effects of IL-8 trout on humoral immunity.

In addition to humoral immunity, accumulating evidence showed that cell-mediated immunity and/or local immunity play important roles in pathogen protection achieved by vaccines, particularly mucosal vaccination in fish [43]. Additionally, several important virus pathogens can cause acute diseases and high mortality and result in persistent infections in salmons [44, 45]. The surviving fish, which may become virus carriers without showing symptoms, potentially pose a threat to the health of naïve fish. Cell-mediated responses play an important role in clearance of virus, but viruses may evade cellular immunity and persist *in vivo* [46]. Data demonstrated that trout IL-2, IL-8, IL-15, IL-1β, or IL-17 all showed conserved abilities to enhance antigen-specific proliferative T-cell responses [47–50]. IL-8 coinjection can also induce considerably strong lymphocyte proliferation in the two test points. IL-1β only functions at early time points. Coinjection of IL-2, IL-15, and IL-17 induces delayed strong lymphocyte proliferation.

Importantly, a correlation was observed between cytokine plasmid enhancement of challenge protection and adaptive immune response. Observed cytokines also enhanced protection by their distinct actions. IL-2 and IL-17 enhanced the majority of tested immune gene expressions, NAb production, longevity, and cell-mediated immunity. Hence, these cytokines can improve protection the most significantly. Previous studies showed that trout IL-8 injection can elicit a marked total number of leukocytes present in the peritoneal cavity [51]. Coinjection of IL-8 with the glycoprotein gene from viral hemorrhagic septicemia virus can also modulate cytokine response in rainbow trout [36]. However, actual adjuvant effects of trout IL-8 on protection are still unknown. According to our results, trout IL-8 can enhance protection mainly through cell-mediated immunity. iIFN1a and IFN-γ2 also exerted limited effects on cell-mediated immune response, but both improved protective efficiency similar to IL-2 and IL-17. Injection with fish IFN plasmid alone can provide protection to some extent [32]. Our previous studies also confirmed antiviral activities of recombinant iIFN1a and IFN-γ2 proteins against IHNV, whereas protection decreases within 3 dpi [24, 25]. Notably, the challenge was performed at 28 dpi. Therefore, we believed that positive effects on protection were not due to their antiviral activities but enhanced NAb response. Nevertheless, adjuvant effect of IL-15 was less clear in challenge experiments because weak modulation of protection showed no good agreement with its comparable stimulated effects. Nonetheless, all cytokines efficiently improve pG DNA vaccination of trout against IHNV-mediated mortality.

In this study, seven types of trout cytokines were evaluated as adjuvants because of their abilities to enhance antigen-specific immune responses following injection delivery. Results showed that these cytokines exerted positive effects on a variety of immune responses and protective efficiency. Additionally, their modulations on immune outcomes depended on cytokine types and were associated with the used dose (at least in terms of different immune relevant gene expressions). However, further studies are required to confirm the responses discussed, especially possible variances existing between the combination with other types of antigen and delivery methods. Nevertheless, our study support that trout cytokines are novel adjuvants, which can also enhance certain immune responses in vaccines.

## MATERIALS AND METHODS

### Ethics statement

Investigations were conducted in accordance with the ethical standards and the Guidelines of European Union Council Directive 2010/63/EU (http://ec.europa.eu/environment/chemicals/lab_animals/legislation_en.htm) for the protection of animals used for scientific purposes. This study was also approved by the Committee of the Ethics on Animal Care and Experiments at Heilongjiang River Fishery Research Institute of Chinese Academy of Fishery Sciences.

### Fish, virus strain, plasmid, and reagent

Healthy rainbow trout (approximately 10 g) were maintained in aerated fiberglass tanks supplied with a continuous flow of recirculating freshwater at 15 °C. Sn1203 strain of IHN virus (GenBank NO.: KC660147.1) was isolated from rainbow trout with clinical symptoms of IHN. Stock titer was 3 × 10^6^ plaque forming units (pfu)/ml. RTG-2 (ATCC CCL-55) cells were cultured in Eagle's minimal essential medium supplemented with 10% fetal bovine serum (FBS, Gibco).

Based on the sequence of glycoprotein gene of IHNV Sn1203, a pair of primers (5′- GAATTCATGGACACCATGATCACCACTCCG-3′ and 5′-GGATCCTCAGGACCGGTTTGCCAGGTGAT-3′; underlined sequences are restriction sites for enzymes *Eco*RI and *Bam*HI) were designed to amplify the glycoprotein gene. cDNA was prepared as PCR template from IHNV. Subsequently, the glycoprotein gene of IHNV Sn1203 was cloned into pEE12.4 plasmid and designated as pG. Recombinant G protein of IHNV was generated in prokaryotic expression system, and its immunogenicity was also confirmed [52]. The anti-IgM antibody was generated based on recombinant truncated IgM of rainbow trout in our previous work [53].

### Cloning and construction of plasmids

According to sequences of IL-2, IL-8, IL-15, IL-17, IL-1β, iIFN1a, and IFN-γ2 (Gen Bank NO. NM_001164065.1, AJ279069.1, AJ555868.1, NM_001124619.2, NM_001124347.2, FJ184370.1, and FM864345.1), primers were designed to amplify their corresponding genes (Table 1). cDNA was prepared as PCR template according to our previous method [24]. PCR products were analyzed by agarose gel electrophoresis and purified using TIANgel Midi Purification Kit (Tiangen, China). Purified DNA fragments and pET32a or pcDNA3.1 vector were digested and linked by T4 DNA ligase to construct recombinant plasmids. Positive clones were identified by colony PCR using the primers indicated above and digested with restriction enzymes. Plasmids from positive clones were extracted using GeneJET Plasmid Miniprep Kit (Thermo Scientific, USA) and subsequent DNA sequencing. Recombinant eukaryotic expression plasmids were extracted using GeneJET Endo-Free Plasmid Maxiprep Kit (Thermo Scientific, USA), quantified, and stored at −20 °C carefully.

### Protein expression, purification, and refolding

Prokaryotic expression plasmids were transformed into Rosetta (DE3) PLysS cells and induced with addition of IPTG (1 mM) when the culture reached between 0.4 and 0.6 at OD_600_. Cells were harvested by centrifuging the culture at 8000 g for 5 min at 4 h after induction. Cell pellets were lysed by sonication. The presence of fusion proteins in supernatant and the pellet containing inclusion bodies were analyzed by 12% sodium dodecyl sulfate polyacrylamide gel electrophoresis (SDS-PAGE). Inclusion bodies were washed twice with 2 M urea solutions and dissolved in 8 M urea solutions. Soluble fractions were purified using protein purification with Ni–NTA protein purification system (Qiagen, USA). Purified proteins were refolded by gradient dialysis from 6 M urea solutions to PBS containing 5% glycerol at 4 °C and analyzed by 12% SDS-PAGE. Finally, refolded proteins were quantified and stored at −80 °C.

### Preparation of polyclonal antibodies

Anti-IL-17 antibodies were produced in our previous work [23]. Twenty-four female BALB/c mice (7 weeks old) were divided into six groups. Prior to immunization, three mice were bled by the tail vein. Afterward, mice from each group were immunized intraperitoneally with 100 μg of refolded proteins mixed with complete freund's adjuvant (CFA) individually. After 1 week, the mice were subsequently boosted thrice with 50 μg of proteins mixed with incomplete freund's adjuvant (IFA) at two-week intervals. Blood samples were obtained from mice tail vein at 1 week after each immunization. Sera were isolated to determine antibody titers by ELISA. At 1 week after the last injection, mice were killed to collect the sera, which were stored at −20 °C. Antiserum titers were determined by indirect ELISA.

### Indirect IFA

An indirect IFA was performed to confirm expression of constructs in RTG-2 cells.. RTG-2 cells were cultured in six-well plates until cell density was 60%–80% confluent. Cells were transfected with 2 μg of plasmids per well using Lipofectamine® 2000 Transfection Reagent (Invitrogen, USA). Cells that were transfected with pcDNA3.1 empty vector served as negative controls. At 48 h after transfection, cells were fixed with 2% paraformaldehyde for 12 min and washed with PBS buffer. Subsequently, cells were incubated with corresponding polyclonal antibodies at 1:100 dilutions at 37 °C for 1 h. The control group was incubated with pool polyclonal antibodies. After three washes with PBS, cells were incubated with FITC-conjugated Goat Anti-Mouse IgG (Abcam, USA) at 37 °C for 1 h. After four washes with PBS, cells were observed under fluorescent microscope.

### Real-time PCR detection

A total of 115 fish were divided into 23 groups, anesthetized with eugenol, and injected i.m. with 2.5 μg of pG alone or pG with cytokine plasmids (0.5, 2.5, or 5 μg). The fish injected with pcDNA3.1 served as negative control. At 3 dpi, spleen tissues from three fish from each experimental group were collected and stored in liquid nitrogen.

Total RNA of samples was extracted with an SV Total RNA Isolation System (Promega, USA) and quantified. cDNA was synthetized from 1 μg of total RNA with a PrimeScript™ RT Master Mix. Relative quantification real-time PCR was performed on an Applied Biosystems 7500 according to manufacturer's instructions for SYBR® Premix Ex Taq™ II (Tli RNaseH Plus). Primers were referenced from publications [54, 55] and are listed in Table 1. Expression level of β-actin served as internal control. Relative expression level was obtained by normalizing the expression of the target gene to that of β-actin. All PCR reactions were performed in triplicate. Relative expression ratio (R) was calculated and compared with those of untreated samples using 2^−ΔΔC^_T_ method [56]. Fold changes in immune gene expression in adjuvant groups were calculated relative to that in pG group.

### Vaccination, sample collection, and challenge

According to immune gene expressions of different combinations of pG and cytokine adjuvants, injection dose of p-cytokines was confirmed. A total of 540 fish were divided into nine groups, anesthetized with eugenol, and injected intramuscularly with pG and pG with cytokine plasmid and pcDNA3.1 plasmid. Serum samples from three individuals in each group were prepared and stored at −20 °C at each week of post immunization until 35 dpi. At 14 and 28 dpi, leukocytes from spleens of three rainbow trout from each experimental group were isolated using Ficoll-Paque PLUS (1.077 and 1.05 g/ml, GE Healthcare, Sweden) according to a previous method [57]. At 28 dpi, the remaining 30 healthy fish from each group were challenged with 50 μl of IHNV (300 pfu) by intraperitoneal injection. The fish were monitored daily for clinical signs of the disease, and dead fish were removed. Survival of each group was recorded daily for 14 days, and cumulative percent survival was determined.

### Detection of specific IgM by indirect ELISA

The 96-well ELISA plates were coated with recombinant G protein at 0.1 μg in carbonate buffer at pH 9.6 with 100 μl per well and incubated overnight at 4 °C. After three washes with 0.05% Tween 20 in PBS buffer (PBST), the plates were blocked with PBST containing 1% gelatin (Sigma-Aldrich) at 37 °C for 1 h. After four washes with PBST, the wells were incubated with 100 μl of serum samples with different dilutions for 1 h. Wells were washed again with PBST for four times and were incubated with 100 μl of anti-IgM antibody at 1:250 dilution for 1 h. After another four washes with PBST, the wells were incubated with horseradish peroxidase-conjugated goat antirabbit IgG antibody (Abcam, USA) at 37 °C for 0.5 h. After the final washes, tetramethyl benzidine solution was added at 70 μl per well. After 12 min, the reaction was stopped with addition of H_2_SO_4_ (1 M). Absorbance was measured at 450 nm using a plate reader (Molecular Devices).

### NAb titers

PNT procedure for IHNV was described previously. Briefly, three serum samples from each experiment group were pooled and heat-inactivated at 45 °C for 30 min. Afterward, the two-fold dilution series of serum (1: 20 to 1: 320) was incubated with an equal volume of IHNV stock for 30 min at 18 °C. Samples were added to RTG-2 cells, which were pretreated with 7% polyethylene glycol and adsorbed after 1 h. Cells were overlaid with a medium containing 4% FBS and 1% methyl cellulose medium (Sigma, USA). When cytopathic effects were observed, cells were fixed with formalin and stained with crystal violet. The highest dilution that resulted in 50% reduction in that of negative control was considered the NAb titer.

### Lymphocyte proliferation assay

Leukocyte suspension was added in triplicate to 96-well plates. Experimental groups were incubated with the medium containing 2 μg/ml recombinant G protein. A total of 5 μg/ml PHA (Sigma, USA) was added to cultures, which were used as positive control. Negative control cells were incubated with the medium. Cell proliferation was assessed using a water-soluble tetrazolium salt-1 Cell Proliferation and Cytotoxicity Assay Kit (Beyotime, China) according to the protocol manual. Results were presented as stimulation index, which was calculated as the ratio of *A*450 of stimulated to that of unstimulated proliferation.

### Statistical analysis

Comparisons of gene expression between adjuvant groups and the vaccine group were performed using one-way ANOVA. Student's t-test was also used to compare some paired samples. Differences were considered statistically significant at P < 0.05.
